# Ki-67 as a Predictor of Metastasis in Adrenocortical Carcinoma: Artificial Intelligence Insights from Retrospective Imaging Data

**DOI:** 10.3390/jcm14144829

**Published:** 2025-07-08

**Authors:** Andrew J. Goulian, David S. Yee

**Affiliations:** 1 College of Medicine, California Northstate University, Elk Grove, CA 95757, USA; 2 Department of Urology and Genitourinary Oncology, Sutter Health, Roseville, CA 95661, USA

**Keywords:** adrenocortical carcinoma, Ki-67, metastasis prediction, artificial intelligence, random forest, risk stratification, minimally invasive surgery, radiomics, precision oncology

## Abstract

**Background/Objectives:** Adrenocortical carcinoma (ACC) is a rare, aggressive malignancy with poor prognosis, particularly in metastatic cases. The Ki-67 proliferation index is a recognized marker of tumor aggressiveness, yet its role in guiding diagnostic imaging and surgical decision-making remains underexplored. This study evaluates Ki-67’s predictive value for metastasis at diagnosis, leveraging artificial intelligence (AI) to inform personalized, minimally invasive strategies for ACC management. **Methods:** We retrospectively analyzed 53 patients with histologically confirmed ACC from the Adrenal-ACC-Ki67-Seg dataset in The Cancer Imaging Archive. All patients had Ki-67 indices from surgical specimens and preoperative contrast-enhanced CT scans. Descriptive statistics, *t*-tests, ANOVA, and multivariable logistic regression evaluated associations between Ki-67, tumor size, age, and metastasis. Random Forest classifiers—with and without the Synthetic Minority Oversampling Technique (SMOTE)—were developed to predict metastasis. A Ki-67-only model served as a baseline comparator. Model performance was assessed using the area under the curve (AUC) and DeLong’s test. **Results:** Patients with metastatic disease had significantly higher Ki-67 indices (mean 39.4% vs. 21.6%, *p* < 0.05). Logistic regression identified Ki-67 as the sole significant predictor (OR = 1.06, 95% CI: 1.01–1.12). The Ki-67-only model achieved an AUC of 0.637, while the SMOTE-enhanced Random Forest achieved an AUC of 0.994, significantly outperforming all others (*p* < 0.001). **Conclusions:** Ki-67 is significantly associated with metastasis at ACC diagnosis and demonstrates independent predictive value in regression analysis. However, integration with machine learning models incorporating tumor size and age significantly improves overall predictive accuracy, supporting AI-assisted risk stratification and precision imaging strategies in adrenal cancer care.

## 1. Introduction

Adrenocortical carcinoma (ACC) is a rare and aggressive malignancy of the adrenal cortex, with an annual incidence of 0.7–2 cases per million and a poor five-year survival rate, particularly in patients with metastatic disease at diagnosis [1,2]. Despite advances in surgical techniques, such as minimally invasive and robotic adrenalectomy, and systemic therapies, early detection and accurate risk stratification remain critical challenges for improving patient outcomes [3,4]. Emerging evidence highlights the prognostic value of comprehensive histopathological assessments, including Ki-67, though challenges persist in standardizing its evaluation across diverse ACC cohorts [5,6]. Additionally, refined surgical strategies and diagnostic innovations, such as urine steroid metabolomics, underscore the need for integrated approaches to enhance risk stratification and early detection [6,7].

Among histopathological markers, the Ki-67 proliferation index has emerged as a key prognostic indicator in ACC, and is consistently linked to tumor recurrence, overall survival, and metastatic potential [8,9,10,11]. Studies have reported Ki-67 cutoffs of 10–20% as predictive of adverse outcomes, underscoring its clinical relevance [9,11].

However, the standalone predictive utility of Ki-67 is constrained by interobserver variability in immunohistochemical scoring, inconsistent tissue sampling, and limited integration into standardized staging systems [12]. Comprehensive evaluations of Ki-67 alongside clinical variables, such as age, tumor size, laterality, or resection margin status, are scarce, with most prior studies focusing on recurrence or survival rather than metastasis at initial diagnosis [13,14]. Moreover, while meta-analyses have established associations between Ki-67 expression and clinical features such as age and tumor size, they do not assess the relative predictive contribution of Ki-67 compared to these variables within multivariable models [15]. To date, comparative predictive analyses evaluating Ki-67 alongside basic clinical features in models specific to ACC remain largely absent from the literature [15,16]. While Ki-67 is linked to recurrence and survival, its role in predicting metastasis at initial ACC diagnosis remains underexplored, particularly in multivariable predictive models. And the increasing availability of open access imaging repositories, such as The Cancer Imaging Archive (TCIA), combined with advanced computational methods, provides an opportunity to validate Ki-67’s predictive capacity through novel analytical approaches [17,18].

Radiomic methodologies have shown promise in predicting Ki-67 expression preoperatively using contrast-enhanced CT imaging, yet translating these predictions into actionable clinical decisions remains challenging [19]. This difficulty arises due to variability in Ki-67 scoring, lack of consensus on clinical cutoff thresholds, and uncertainty about how Ki-67 should guide imaging surveillance or treatment decisions, such as adjuvant therapy or surgical planning [12,19]. Integrating Ki-67-based predictions with imaging and interventional strategies could optimize preoperative risk assessment and guide minimally invasive procedures in ACC management [20,21].

Recent advancements in artificial intelligence (AI) and machine learning offer significant potential to enhance diagnostic precision and personalize risk stratification in oncology [22,23]. Ensemble learning algorithms, such as Random Forest classifiers paired with resampling techniques like the Synthetic Minority Oversampling Technique (SMOTE), provide robust classification capabilities, particularly for the imbalanced datasets common for rare cancers like ACC [24,25,26]. These methods address the limitations of traditional statistical models, which often lack power due to small sample sizes [25].

In this study, we analyzed clinical and histopathological data from the publicly accessible Adrenal-ACC-Ki67-Seg dataset on TCIA [18]. Our primary objective was to evaluate the Ki-67 index’s predictive capacity for metastatic disease at initial diagnosis and assess its incremental value when combined with tumor size and age, using both logistic regression and Random Forest machine learning techniques. We aim to develop data-driven, reproducible risk stratification models for ACC, contributing to multimodal approaches that integrate imaging, histopathological, and computational tools to guide minimally invasive strategies in urological oncology [20,21].

## 2. Materials and Methods

### 2.1. Study Design

This retrospective study utilized de-identified clinical and imaging data obtained from The Cancer Imaging Archive (TCIA), a publicly funded resource supported by the National Cancer Institute (Bethesda, MD, USA) and maintained by the Department of Biomedical Informatics within the College of Medicine at the University of Arkansas for Medical Sciences (UAMS) (Little Rock, AR, USA) in collaboration with the UAMS Information Technology department and the Department of Biomedical Informatics at Emory University (Atlanta, GA, USA).As all TCIA data are de-identified in compliance with the HIPAA Safe Harbor Method, and the de-identification process is conducted under protocols approved by the Institutional Review Board of the hosting institution, this study was exempt from additional IRB review and the requirement for informed consent was waived [17].

Clinical and imaging data were extracted from the Adrenal-ACC-Ki67-Seg dataset hosted on TCIA [18]. Patients were included if they met the following criteria: a confirmed diagnosis of adrenocortical carcinoma (ACC); underwent surgical resection of the tumor; Ki-67 index determined from histopathological analysis of the resected specimen; and had available contrast-enhanced abdominal CT imaging performed prior to surgery. Patients were excluded if the Ki-67 index was determined via biopsy rather than resected tumor tissue, based on prior studies indicating that whole-tumor evaluation provides more reliable quantification [18].

A total of 53 patients diagnosed with ACC between 2006 and 2018 were included. Among them, 7 patients (13.2%) had metastatic disease at initial diagnosis, while 46 (86.8%) did not. The dataset included clinical, demographic, and histopathologic variables such as age, sex, race, tumor laterality, tumor size (in cm), Ki-67 index (%), resection margin status, T and N staging, and time from imaging to diagnosis (in days). All patients underwent surgical resection, and Ki-67 indices were quantified from the full tumor specimen.

### 2.2. Statistical Analysis

All statistical analyses were conducted in R version 4.5.0. Descriptive statistics, including means, standard deviations (SDs), medians, and counts, were calculated for all clinical and histopathologic variables. Variables were stratified by metastatic status at diagnosis (yes vs. no) to facilitate group-level comparisons.

For continuous variables including age, tumor size, Ki-67 index, and days from imaging to diagnosis, group comparisons were performed using Welch’s two-sample *t*-tests. One-way analysis of variance (ANOVA) was used to assess differences in the Ki-67 index across categories of resection margin, laterality, T stage, N stage, sex, and race. Pearson correlation was used to evaluate the association between Ki-67 index and tumor size.

A chi-square test was performed to assess the association between resection margin status and metastasis at diagnosis. A multivariable linear regression model was constructed to evaluate the relationship between Ki-67 index and potential predictors, including tumor size, age, and N stage.

Finally, a multivariable logistic regression model was constructed using age, tumor size, and Ki-67 index to explore their joint association with metastatic disease at diagnosis. This reduced model was chosen based on clinical relevance and model stability [27]. A separate full model including all available predictors was attempted but failed to converge due to complete separation among certain categorical features. All statistical tests were two-sided, and *p*-values < 0.05 were considered statistically significant.

### 2.3. Artificial Intelligence

To develop a clinically relevant predictive model of metastatic disease at diagnosis, we trained supervised classifiers using the Random Forest algorithm [28]. This approach was selected due to its resilience to multicollinearity, ability to model nonlinear interactions, and effectiveness in small-to-moderate sample sizes typical of rare cancer datasets [24,26].

Ten predictor variables were used: age, sex, race, tumor laterality, tumor size (cm), resection margin status, T stage, N stage, Ki-67 index (%), and days from imaging to diagnosis. All categorical variables were one-hot encoded, and the dataset was complete with no missing values [29].

Given the moderate class imbalance (7 metastatic vs. 46 non-metastatic cases), the Synthetic Minority Oversampling Technique (SMOTE) was employed to synthetically balance the dataset prior to model training. SMOTE interpolates new examples from the minority class to improve classifier performance and mitigate overfitting [26].

Model training was conducted using repeated 10-fold cross-validation with five repetitions. Random Forest models were trained with 500 trees, and Gini impurity was used as the splitting criterion. Model performance was primarily assessed using the area under the receiver operating characteristic curve (AUC-ROC). Secondary performance metrics included sensitivity, specificity, and balanced accuracy. Variable importance was computed using permutation-based importance scores from the SMOTE-enhanced Random Forest model [30]. To directly assess the standalone predictive performance of Ki-67, we trained an additional Random Forest model using Ki-67 as the sole predictor. The same cross-validation strategy and performance metrics were applied as in the multivariable models. This single-variable model served as a baseline comparator to evaluate whether the inclusion of additional clinical and pathological features meaningfully improved discrimination.

### 2.4. Model Performance Comparison

To statistically compare the discriminatory performance of each classifier, we conducted DeLong’s test for paired receiver operating characteristic (ROC) curves. Pairwise comparisons were performed between the logistic regression model, the Random Forest model trained without class balancing, and the Random Forest model trained on SMOTE-balanced data. This nonparametric method evaluates whether observed differences in area under the ROC curve (AUC) are statistically significant, accounting for the paired nature of predictions on the same dataset [31]. All AUC comparisons were two-sided, with *p*-values less than 0.05 considered statistically significant.

### 2.5. Model Evaluation Metrics

For each model, we evaluated discrimination using the area under the receiver operating characteristic curve (AUC). Optimal thresholds were determined using Youden’s Index. To estimate model performance at the chosen threshold, we calculated sensitivity, specificity, positive predictive value (PPV), and negative predictive value (NPV).

To derive 95% confidence intervals (CIs) for sensitivity, specificity, PPV, and NPV, we employed a nonparametric bootstrap procedure with 1000 resamples [32]. For each bootstrap iteration, predictions and true labels were sampled with replacement, binary predictions were generated at the fixed threshold from Youden’s Index, and the metric of interest was calculated. Final point estimates represent the mean across all iterations, with the 2.5th and 97.5th percentiles used to construct the confidence intervals [33]. This approach allowed for robust interval estimation across all four models: logistic regression, random forest without SMOTE, random forest with SMOTE, and random forest using Ki-67 alone.

## 3. Results

### 3.1. Descriptive Characteristics

This study included 53 patients with histopathologically confirmed adrenocortical carcinoma (ACC), each of whom underwent surgical resection. The study aimed to assess whether Ki-67 index and other clinical and pathological variables could predict the presence of metastasis at diagnosis.

Among the cohort, 7 patients (13.2%) presented with metastatic disease, and 46 (86.8%) were non-metastatic at the time of diagnosis. The mean age was 52.8 years (SD: 13.5), and 58.5% of the cohort were female.

The mean tumor size was 11.5 cm (SD: 6.5), ranging from 3.2 to 33.0 cm. Patients with metastatic disease tended to have slightly smaller tumors (mean: 9.4 cm) compared to those without metastasis (mean: 11.9 cm). The mean Ki-67 proliferation index was 23.9% (SD: 18.4), with significantly higher levels in metastatic patients (mean: 39.4%) than in non-metastatic patients (mean: 21.6%).

In terms of resection margins, 35 patients (66.0%) had R0 (negative) margins, 10 (18.9%) had R1 (microscopically positive) margins, and 8 (15.1%) had RX margins. The RX category indicates cases in which the resection margin status was not clearly documented in the pathology report and was therefore classified as unknown. Most tumors were right-sided (45%) or left-sided (55%), with no significant laterality difference between groups.

T-stage distribution favored T2 and T3 tumors overall. Nodal staging revealed N1 disease in 4 of 7 metastatic patients (57.1%) compared to just 1 of 46 non-metastatic patients (2.2%). Descriptive characteristics stratified by metastasis status are summarized below in Table 1.

### 3.2. Analysis Association Between Ki-67 and Clinical Variables

To assess whether the Ki-67 proliferation index was associated with demographic or anatomical variables, a multivariable linear regression model was constructed that included tumor size, patient age, and N stage as predictors. The model was not statistically significant overall (adjusted R^2^ < 0), and none of the included variables showed a significant association with Ki-67 expression (*p* > 0.05 for all). This suggests that Ki-67 may reflect a biologically distinct feature of tumor aggressiveness, independent of tumor size or patient age.

Pearson correlation analysis revealed no significant relationship between tumor size and Ki-67 index (r = −0.02, *p* = 0.90), further supporting the independence of proliferative activity from anatomical tumor burden.

### 3.3. Comparison of Ki-67 Index by Metastasis and Other Clinical Features

**Figure 1:**
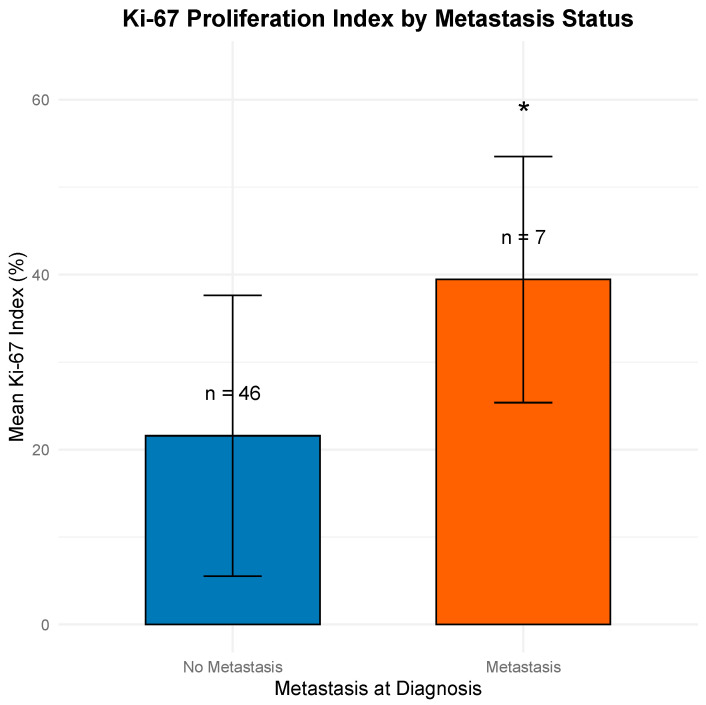
Mean Ki-67 proliferation index in patients with and without metastasis at diagnosis. Patients with metastatic disease had significantly higher Ki-67 indices (mean 39.4%, SD 14.1%) than those without metastasis (mean 21.6%, SD 16.1%) (*p* = 0.014, Welch’s *t*-test). Bars represent means ± 1 standard deviation. Note: * *p* < 0.05 indicates a statistically significant difference.

A Welch’s two-sample *t*-test demonstrated that patients with metastatic disease at diagnosis had significantly higher Ki-67 indices (mean: 39.4%) compared to non-metastatic patients (mean: 21.6%) (*p* = 0.014). This finding supports Ki-67 as a potential marker of systemic disease at presentation. Figure 1 illustrates the distribution of Ki-67 by metastatic status.

One-way ANOVA showed no statistically significant differences in Ki-67 expression by resection margin status (*p* = 0.703), laterality (*p* = 0.925), T staging (*p* = 0.76), N staging (*p* = 0.484), sex (*p* = 0.821), or race (*p* = 0.457). A chi-square test examining the association between resection margin status and metastasis approached statistical significance (*p* = 0.076), suggesting a potential trend that warrants further investigation in larger cohorts.

### 3.4. Logistic Regression Model for Metastasis

A multivariable logistic regression model including the Ki-67 index, tumor size, and age was constructed to evaluate predictors of metastatic disease at diagnosis. Among these, only Ki-67 was statistically significant (OR = 1.06, 95% CI: 1.01–1.12, *p* < 0.05), indicating that each one percent increase in Ki-67 was associated with a six percent increase in the odds of presenting with metastasis. Tumor size and age were not significant predictors (*p* > 0.05). This association is visually supported by Figure 1, which illustrates markedly higher Ki-67 indices in patients with metastatic disease. The logistic model demonstrated good discriminatory performance with an AUC of 0.84.

A full logistic regression model including all available predictors was attempted but failed to converge due to quasi-complete separation in several categorical variables, consistent with limitations in small-sample binary modeling.

### 3.5. Random Forest Classifier with and Without SMOTE

To improve prediction and account for class imbalance, a supervised classification model was trained using Random Forest. The final model incorporated ten predictors: age, sex, race, tumor laterality, tumor size, resection margin, T stage, N stage, Ki-67 index, and time from imaging to diagnosis. Categorical variables were one-hot encoded [27].

A Random Forest model trained without oversampling achieved an AUC of 0.793, sensitivity of 57.2%, and specificity of 83.6%. After applying SMOTE, performance improved substantially, with an AUC of 0.994, sensitivity of 94.3%, and specificity of 97.4%. In comparison, the logistic regression model yielded an AUC of 0.722, sensitivity of 57.2%, and specificity of 83.6%.

To further evaluate whether the inclusion of clinical and pathological variables improved model performance beyond Ki-67 alone, we trained a baseline Random Forest model using only the Ki-67 index. This single-variable Ki-67 model yielded an AUC of 0.660, with a sensitivity of 37.4% and specificity of 78.9%. These results demonstrate that Ki-67 alone provides limited predictive accuracy, supporting the added discriminatory value of including features such as staging, resection margin, and tumor laterality alongside Ki-67.

**Figure 2:**
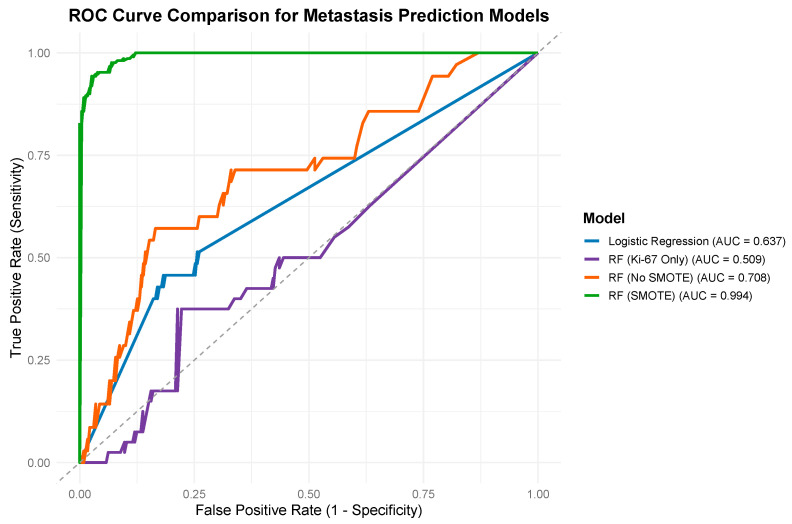
Receiver operating characteristic (ROC) curves for metastasis prediction using four models: Logistic Regression (blue), Random Forest using Ki-67 only (purple), Random Forest without SMOTE (orange), and Random Forest with SMOTE (green). The SMOTE-enhanced Random Forest model achieved the highest AUC (0.994), followed by RF without SMOTE (0.708), Logistic Regression (0.637), and RF with Ki-67 alone (0.509). DeLong tests showed statistically significant improvements in AUC for the SMOTE-enhanced Random Forest model over RF without SMOTE, logistic regression, and the Ki-67-only model (*p* < 0.001 for all). RF without SMOTE also outperformed the Ki-67-only model (*p* < 0.05), whereas its difference from logistic regression was not statistically significant (*p* = 0.297). The diagonal dashed line represents the line of no-discrimination (AUC = 0.5), corresponding to random chance.

Receiver operating characteristic (ROC) curves for all four models are displayed in Figure 2. Pairwise DeLong tests confirmed that the SMOTE-enhanced Random Forest significantly outperformed all other models, including the non-SMOTE Random Forest (*p* < 0.001), logistic regression (*p* < 0.001), and the Ki-67-only model (*p* < 0.001). The non-SMOTE Random Forest significantly outperformed the Ki-67-only model (*p* < 0.05), but not the logistic regression model (*p* = 0.297). The performance difference between logistic regression and the Ki-67-only model approached statistical significance (*p* = 0.062), suggesting marginally better discrimination by the logistic model. All model performance metrics, including AUC variability, PPV, and NPV, are summarized in Table 2.

**Figure 3:**
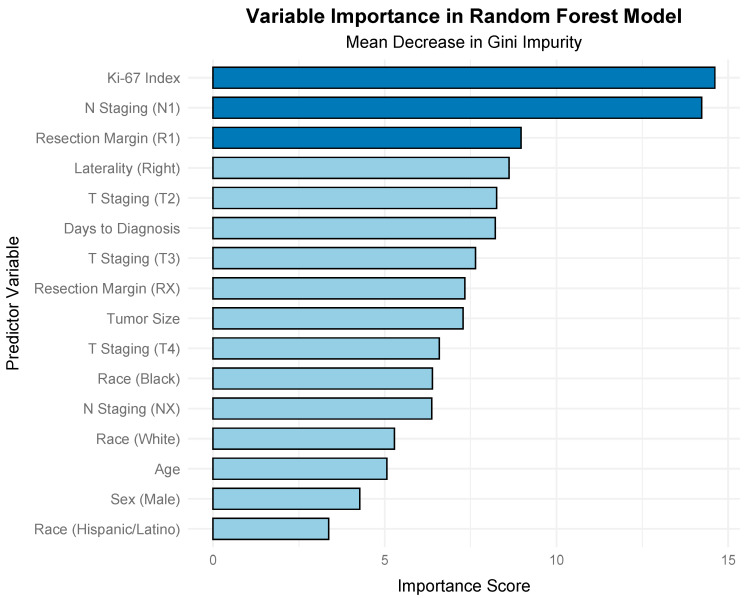
Variable importance in a SMOTE-Enhanced Random Forest Model for Metastasis Prediction. The bar plot displays the mean decrease in Gini impurity for each predictor, with the Ki-67 index showing the highest importance, followed by N staging (N1), resection margin (R1), laterality (right), and T staging (T2). Lower importance scores were observed for variables such as tumor size, age, sex, and race. The top three predictors are shown in a darker shade to highlight their relatively greater contribution to the model.

The SMOTE-enhanced Random Forest model not only improved predictive performance but also provided insights into the relative importance of predictors. As shown in Figure 3, the Ki-67 index was the most influential variable, contributing the highest mean decrease in Gini impurity, followed by N staging (N1), resection margin (R1), laterality (right), and T staging (T2). Variables such as tumor size, age, sex, and race had lower importance scores, indicating a lesser role in distinguishing metastatic from non-metastatic cases. These findings underscore the critical role of Ki-67 as a marker of metastatic potential in this cohort.

## 4. Discussion

### 4.1. Principal Findings

This study underscores the pivotal role of the Ki-67 proliferation index in predicting metastatic disease at the time of adrenocortical carcinoma (ACC) diagnosis, offering a transformative approach to risk stratification through the integration of statistical and machine learning methodologies. Analysis of the dataset revealed that Ki-67 is a dominant biomarker for identifying patients at high risk of metastasis, surpassing the predictive utility of clinical variables such as tumor size and patient age [18,27]. Traditional logistic regression confirmed Ki-67’s independent association with metastatic status, while a SMOTE-enhanced Random Forest model demonstrated exceptional discriminatory power, highlighting Ki-67’s primacy among predictors [34,35,36]. This dual approach not only validates Ki-67’s prognostic significance but also showcases the potential of advanced computational techniques to refine clinical decision-making in rare cancers [37,38]. These findings pave the way for personalized ACC management, enabling tailored imaging surveillance and minimally invasive interventional strategies to optimize patient outcomes [21].

### 4.2. Comparison with Prior Literature

Previous research has consistently identified the Ki-67 proliferation index as a critical prognostic marker in adrenocortical carcinoma (ACC), with thresholds of 10–20% linked to increased recurrence and reduced survival [8,9,11,14,39]. Studies have shown that Ki-67 levels ≥10% are associated with poorer outcomes in localized ACC following surgical resection [10], while its histoprognostic significance extends to oncolytic adrenal tumors [14]. Additionally, Ki-67’s prognostic impact has been demonstrated across both adult and pediatric ACC populations, as well as in other endocrine neoplasms, underscoring its broader relevance [11,39]. However, these studies predominantly employed Cox regression or Kaplan–Meier analyses, focusing on long-term outcomes like recurrence or overall survival rather than metastasis at initial diagnosis [13].

In contrast, radiomic approaches have explored preoperative prediction of Ki-67 expression using contrast-enhanced CT imaging, achieving moderate accuracy but facing challenges in clinical application due to variability in Ki-67 scoring and lack of standardized cutoff thresholds [19]. The present study advances this field by specifically evaluating Ki-67’s predictive capacity for metastatic disease at ACC presentation. By employing a Random Forest classifier with Synthetic Minority Oversampling Technique (SMOTE) to address class imbalance (7 metastatic vs. 46 non-metastatic cases), our approach integrates clinical variables such as age and tumor size, enhancing risk stratification beyond traditional statistical models [24,38].

A key barrier to translating Ki-67-based predictions into clinical practice is the variability in immunohistochemical scoring, driven by inconsistent cutoffs and differences in morphometric techniques [12,19]. This variability limits the integration of Ki-67 into standardized imaging or interventional protocols. Our study mitigates these challenges by leveraging machine learning to achieve exceptional predictive accuracy, providing a robust framework to inform imaging surveillance strategies and guide minimally invasive interventions, such as robotic adrenalectomy, for high-risk ACC patients [21]. This data-driven approach marks a significant step toward precision oncology, distinguishing our work from prior efforts focused on prognostic rather than predictive applications of Ki-67.

### 4.3. Methodological Considerations

The inclusion of tumor size and age alongside Ki-67 in predictive models, despite their non-significant associations with metastasis, was guided by their clinical relevance in ACC staging and surgical planning [3,13]. Incorporating these routinely collected variables allowed us to evaluate Ki-67’s incremental predictive value and ensured model robustness across diverse clinical contexts [27]. Pearson correlation analysis confirmed no significant relationship between Ki-67 and tumor size (r = −0.02, *p* = 0.90) or age (*p* > 0.05), supporting Ki-67’s independent biological role as a marker of tumor aggressiveness [35].

To address the class imbalance in the dataset (7 metastatic vs. 46 non-metastatic cases), the Synthetic Minority Oversampling Technique (SMOTE) was applied, improving the Random Forest model’s area under the receiver operating characteristic curve (AUC) from 0.793 with 95% CI (0.726–0.861) to 0.994 with 95% CI (0.990–0.998) across 10-fold cross-validation repeated five times (*p* < 0.001, DeLong’s test) [24,35,37]. A non-SMOTE Random Forest model was also evaluated to confirm that SMOTE’s resampling, rather than the Random Forest algorithm alone, drove the performance gain [37]. The Random Forest classifier was constructed with 500 trees, using Gini impurity as the splitting criterion and no constraints on tree depth to maximize flexibility given the small sample size [37]. AUC was selected as the primary performance metric due to its robustness in evaluating binary classifiers on imbalanced datasets [36]. To capture clinical relevance, secondary metrics included sensitivity, specificity, positive predictive value (PPV), and negative predictive value (NPV), each reported with bootstrapped 95% confidence intervals. This approach provides a comprehensive and reliable assessment of model performance variability across key diagnostic thresholds [40].

### 4.4. Clinical Implications

Our findings highlight Ki-67’s integrative role in optimizing imaging and interventional strategies for ACC. Patients with high Ki-67 indices (≥20%) are at elevated risk of metastasis, warranting intensified preoperative imaging, such as PET-CT or contrast-enhanced CT, to detect occult metastatic disease and guide surgical planning [9,18]. Postoperatively, Ki-67 can inform surveillance protocols, with high-risk patients benefiting from more frequent imaging to monitor for recurrence [17]. In terms of interventions, identifying high-risk patients early enables tailored approaches, such as minimally invasive or robotic adrenalectomy, which reduce morbidity compared to open surgery [21]. Moreover, patients with elevated Ki-67 may benefit from early adjuvant therapies, such as mitotane, radiation therapy, or enrollment in clinical trials, to improve prognosis [4,9]. The superior performance of the SMOTE-enhanced Random Forest model suggests that machine learning can augment traditional staging systems, enabling data-driven patient stratification [12,23]. This approach aligns with precision oncology by integrating histopathological data with clinical decision-making, potentially reducing unnecessary interventions in low-risk patients while prioritizing aggressive management in high-risk cases [41,42].

### 4.5. Limitations

The study’s sample size (*n* = 53) limits its generalizability, though SMOTE and cross-validation mitigated overfitting [24]. Variability in Ki-67 scoring across institutions may introduce measurement error, necessitating standardized protocols [12]. While the Adrenal-ACC-Ki67-Seg dataset includes preoperative CT imaging, we did not incorporate radiomic features, which could enhance predictive accuracy [19,20]. External validation in larger, multi-institutional cohorts is needed to confirm our findings before clinical adoption [43].

### 4.6. Future Directions

Future research should validate Ki-67’s predictive role in diverse cohorts and integrate it with radiomic and genomic data to develop multimodal models [8,41,44]. For the Special Issue, combining Ki-67 with imaging biomarkers from contrast-enhanced CT could improve preoperative risk assessment, guiding the selection of minimally invasive techniques [20]. Longitudinal studies examining Ki-67’s association with treatment response and recurrence will further elucidate its role across the ACC continuum [4]. Ultimately, integrating histologic, radiomic, and molecular data into AI-driven models could personalize ACC management, optimizing imaging, interventions, and adjuvant therapies [12,23].

## 5. Conclusions

The Ki-67 proliferation index is a powerful, independent predictor of metastatic disease at ACC diagnosis, offering actionable insights for imaging and interventional strategies. Its significant association with metastasis (OR = 1.06, *p* < 0.05) and dominance in the SMOTE-enhanced Random Forest model (AUC = 0.994) highlight its potential to be incorporated and further refine risk stratification beyond traditional markers like tumor size and age [36,38]. For the Special Issue, Ki-67 can guide intensified imaging surveillance such as PET-CT for high-risk patients and inform the selection of minimally invasive or robotic adrenalectomy to minimize morbidity [18,21]. Early identification of high Ki-67 levels (≥20%) also supports timely initiation of adjuvant therapies, such as mitotane or radiation, and enrollment in clinical trials to improve outcomes [4,9].

Machine learning enhances predictive accuracy, with the SMOTE-enhanced Random Forest model significantly outperforming logistic regression (*p* < 0.001), highlighting its utility in rare cancers like ACC [37]. The model’s high sensitivity (94.3%) and specificity (97.4%) support its potential clinical applicability for early risk stratification, aligning with the goals of precision oncology [41]. Future models integrating Ki-67 with radiomic and genomic data could further personalize care, optimizing imaging protocols and interventional techniques [20,44]. By reimagining how biomarkers like Ki-67 are integrated into clinical pathways, this study paves the way for data-driven, minimally invasive management of ACC, potentially transforming patient outcomes.

## Figures and Tables

**Table 1:** Baseline demographic, clinical, and tumor characteristics of 53 patients with adrenocortical carcinoma (ACC), stratified by the presence of metastatic disease at diagnosis. Values are reported as mean ± standard deviation for continuous variables and counts with percentages for categorical variables.

Variable	Non-Metastatic (*n* = 46)	Metastatic (*n* = 7)	Total (*n* = 53)
Age, years (mean ± SD)	53.4 ± 13.6	48.9 ± 13.2	52.8 ± 13.5
Sex, *n* (%)			
Female	27 (58.7)	4 (57.1)	31 (58.5)
Male	19 (41.3)	3 (42.9)	22 (41.5)
Race, *n* (%)			
White	36 (78.3)	5 (71.4)	41 (77.4)
Black	3 (6.5)	1 (14.3)	4 (7.5)
Hispanic or Latino	5 (10.9)	1 (14.3)	6 (11.3)
Asian	2 (4.3)	0 (0.0)	2 (3.8)
Laterality, *n* (%)			
Right	19 (41.3)	5 (71.4)	24 (45.3)
Left	27 (58.7)	2 (28.6)	29 (54.7)
Tumor Size, cm (mean ± SD)	11.9 ± 6.8	9.4 ± 2.8	11.5 ± 6.5
Ki-67 Index, % (mean ± SD)	21.6 ± 16.1	39.4 ± 14.1	23.9 ± 18.4
Resection Margin, *n* (%)			
R0 (Negative)	33 (71.7)	2 (28.6)	35 (66.0)
R1 (Positive)	7 (15.2)	3 (42.9)	10 (18.9)
RX (Unknown)	6 (13.0)	2 (28.6)	8 (15.1)
T Staging, *n* (%)			
T1	4 (8.7)	0 (0.0)	4 (7.5)
T2	20 (43.5)	0 (0.0)	20 (37.7)
T3	19 (41.3)	5 (71.4)	24 (45.3)
T4	3 (6.5)	2 (28.6)	5 (9.4)
N Staging, *n* (%)			
N0	43 (93.5)	3 (42.9)	46 (86.8)
N1	1 (2.2)	3 (42.9)	4 (7.5)
NX	2 (4.3)	1 (14.3)	3 (5.7)
Days to Diagnosis (mean ± SD)	34.7 ± 41.7	34.9 ± 17.1	34.7 ± 39.4

**Table 2:** Performance metrics of predictive models for metastasis in ACC. Each model’s discrimination ability is summarized using area under the ROC curve (AUC), along with sensitivity, specificity, positive predictive value (PPV), and negative predictive value (NPV), all reported with bootstrapped 95% confidence intervals. The models evaluated include logistic regression, Random Forest using only Ki-67 as a predictor, Random Forest without class balancing, and Random Forest with SMOTE. The SMOTE-enhanced Random Forest demonstrated superior predictive performance across all metrics. Conversely, the Ki-67 only model showed limited discrimination, emphasizing the benefit of integrating additional clinical and pathological variables.

Model	AUC (95% CI)	Sensitivity % (95% CI)	Specificity % (95% CI)	PPV (95% CI)	NPV (95% CI)
Logistic Regression	0.722 (0.644–0.790)	0.458 (0.286–0.625)	0.818 (0.767–0.869)	0.275 (0.162–0.385)	0.909 (0.867–0.945)
RF (Ki-67 Only)	0.660 (0.577–0.743)	0.374 (0.229–0.531)	0.789 (0.734–0.845)	0.240 (0.138–0.343)	0.876 (0.829–0.923)
RF (no SMOTE)	0.793 (0.726–0.861)	0.572 (0.405–0.731)	0.836 (0.788–0.882)	0.345 (0.229–0.468)	0.928 (0.892–0.959)
RF + SMOTE	0.994 (0.990–0.998)	0.943 (0.911–0.972)	0.974 (0.951–0.991)	0.971 (0.946–0.990)	0.949 (0.920–0.975)

## Data Availability

The data supporting the findings of this study are publicly available through The Cancer Imaging Archive (TCIA) under the dataset entitled Adrenal-ACC-Ki67-Seg | Voxel-level segmentation of pathologically-proven Adrenocortical carcinoma with Ki-67 expression. The dataset can be accessed via the following DOI: https://doi.org/10.7937/1FPG-VM46 [18].
